# Florfenicol pharmacokinetics following intravenous and oral administrations and its elimination after oral and bath administrations in common carp *(Cyprinus carpio)*

**Published:** 2017-12-15

**Authors:** Abdolhossein Jangaran Nejad, Rahim Peyghan, Hossein Najafzadeh Varzi, Ali Shahriyari

**Affiliations:** 1 *Department of Clinical Sciences, Faculty of Veterinary Medicine, Shahid Chamran University of Ahvaz, Ahvaz, Iran;*; 2 *Department of Basic Sciences, Faculty of Veterinary Medicine, Shahid Chamran University of Ahvaz, Ahvaz, Iran.*

**Keywords:** Antibiotic, Common carp Florfenicol, Pharmacokinetics

## Abstract

The aim of this study was to evaluate pharmacokinetic profiles of florfenicol after a single dose of intravenous (5.00 mg kg^-1^ body weight) and oral (40.00 mg kg^-1^ body weight) administrations in common carp (*Cyprinus carpio*). The residue depletion of florfenicol was also investigated after oral administration (10.00 mg kg^-1^ body weight) and bath treatment (5.00 mg L^-1^) for 10 consecutive days. Pharmacokinetics of florfenicol in plasma after a single dose administration, at 10 time points (0.50, 1, 2, 4, 8, 12, 24, 72, 120 and 168 hr) and florfenicol concentrations in tissues (plasma, liver and muscle) at three time points (1, 7 and 14 days) after 10 consecutive days, were analyzed by high performance liquid chromatography. The peak concentration of florfenicol was 137.02 ng mL^-1^ and the time to reach peak concentration in plasma was two hr. The elimination half-lives, the volume of distribution at steady state and total body clearance were estimated as 21.40 hr, 0.30 and 0.03 L hr^-1^, respectively. After drug administration for 10 days, it's concentration in plasma and muscle in oral treatment was significantly more than bath treatment in all days. Drug concentrations in the liver after bath treatment were significantly higher for a shorter period than the concentration in the oral treatment, indicating that higher levels of florfenicol for a longer period can be achieved in the tissues after oral drug administration. According to pharmacokinetic results, florfenicol may be a suitable candidate for the treatment of common bacterial infections in common carp farming.

## Introduction

Florfenicol is a broad-spectrum, primarily bacterio-static antibiotic with a range of activity similar to that of chloramphenicol. It is a fluorinated derivative of thiamphenicol effective against many Gram-positive and Gram-negative organisms.^1^ The mechanism of action of this antibiotic involves the inhibition of protein synthesis by binding to the 50S ribosomal subunit of susceptible pathogens. Although it belongs to the family of agents that includes thiamphenicol and chloramphenicol, the structural modifications of florfenicol result in greater resistance to deactivation by bacteria. Because it has proven clinically effective in controlling fish pathogens, florfenicol has been approved for use in Europe, Norway, USA, Canada, Japan, China and South Korea for a variety of fish species.^2^

Pharmacokinetic profiles of florfenicol have been described in Atlantic salmon (*Salmo salar*);^3^^,^^4^ Korean catfish (*Silurus asotus*);^5^ koi carp (*Cyprinus carpio*);^6^ cod (*Gadus morhua*);^7^ red pacu (*Piaractus brachypomus*);^8^ tilapia (*Oreochromis spp*);^9^^-^^11^ crucian carp (*Carassius auratus cuvieri*);^12^ olive flounder (*Paralichthys olivaceus*);^13^ channel catfish (*Ictalurus punctatus*);^14^ yellow catfish, (*Pelteobagrus fulvidraco*)^15^ and rainbow trout, (*Onchorhyncus mykiss*).^16^ Some species of bacteria such as *Aeromonas* and *Flavobacter* can easily infect common carp during some stage of culture, having profound impacts on productivity. It is necessary to treat with antibacterials in order to reduce the mortality rate due to bacterial infection. It is significant that information is available from studies on pharmacokinetics in common carp. This will help to improve treatment efficacy and establish correct dosage regimes and furthermore, it helps to minimize environmental impacts. The aim of the present work was to obtain information on the pharmacokinetics, tissue distribution and elimination of florfenicol following single intravenous and oral doses in common carp. In addition, concentrations of florfenicol in plasma, muscle, and liver were evaluated after oral and bath medication routes for 10 consecutive days. Therefore, the present study will present information on florfenicol distributions in muscle, liver, and plasma and help to determine florfenicol withdrawal period for the common carp.

## Materials and Methods

**Chemicals. **Florfenicol standard was purchased from Sigma Chemicals Co. (St. Louis, USA). Acetonitrile, ethylacetate, high performance liquid chromatography (HPLC) grade hexane and methanol were purchased from Merck (Darmstadt, Germany). Stock standard solutions of florfenicol were prepared as 1 mg mL^-1^ and 0.50 mg mL^-1^ by dissolving the drug in methanol respectively and stored at –20 ˚C.

**Chromatographic conditions. **The analyses were performed on a HPLC system (SPD-10AVP, Shimadzu, Tokyo, Japan) consisting UV-Vis detector (set at 223 nm) and class-VP software. A C18RS 250 by 4.50 mm reverse-phase column was used. The mobile phase of acetonitrile–water (25:75, v/v) with 1.50 mL min^-1^ flow rate was used. The column was operated at 30 ˚C.

**Fish. **One hundred and fifty-five common carp (body weight 100 ± 15.00 g) with no prior history of exposure to florfenicol were purchased from an aquaculture farm in Ahvaz (Khuzestan province, Iran). They were reared in 300 L tanks which were disinfected one day prior to transmission. Important water quality parameters such as temperature (25 - 26 ˚C), pH 7.20 - 7.80 and oxygen (6.00 - 6.50 mg L^-1^) were frequently monitored. The fish were fed drug-free commercial pellet in an amount of 3.00% of the body weight for two weeks. After the acclimation period, the fish were starved for one day before administration of the drug.

**Experimental design. **Fish were randomly assigned to two groups, one for pharmacokinetics and the other for residue depletion analysis. For pharmacokinetics evaluation, in 55 fish, a single dose of intravenous (IV) injections (5.00 mg kg^-1^) and in 55 fish, oral administrations (40.00 mg kg^-1^) by gavages were applied. After 0.50, 1, 2, 4, 8, 12, 18, 24, 72, 120 and 168 hr, fish were sacrificed and blood samples were collected from five fish at each time point. To study the tissue residues of florfenicol, 45 fish were given daily oral doses of 10 mg kg^-1^ and bath doses of 5.00 mg L^-1^ florfenicol for 10 consecutive days. Then, tissue samples (plasma, muscle, and liver) were collected from 15 fish at 1, 7 and 14 days after the end of the experiment. All samples were immediately frozen and stored at –20 ˚C until analyses. 

**Drug administration. **Florfenicol powder was dissolved in a minute quantity of propylene glycol at room temperature using magnetic stirring apparatus and adjusted with distilled water to the final concentrations of 5.00 mg mL^-1^ IV, 2.00 mg mL^-1^ oral administration. Each fish was weighed in a small tank of water and injected with 0.10 mL (corresponding to 5.00 mg kg^-1^ body weight) florfenicol solution into the caudal vein. The position of the needle in the caudal vein was confirmed by aspirating blood into the syringe prior to and after injection. If the needle had dislocated during injection or the fish was heavily bleeding, the fish was excluded from the study and replaced. Individual fish was manually restrained and administered by an intra-gastric method with 2.00 mL florfenicol solution (40.00 mg kg^-1^ body weight) with a gavage tube. In order to preparation of oral suspension for 10 consecutive days, florfenicol powder was dissolved in propylene glycol (2.50 mg mL^-1^). Medicated feed was prepared by blending the drug in the feed (10.00 mg per g of feed). The fish were fed prepared food for 10 consecutive days.

**Sample preparation. **Florfenicol extraction and analysis were carried out according to the procedure described by Feng *et al*.^17^ Plasma sample (1 mL) was added to 15.00 mL graduated plastic-stoppered centrifuge tubes. Each sample was mixed for 2 min and then, 4.0 mL ethyl acetate was added to each tube to precipitate proteins. The supernatant was removed and transferred into another 15.00 mL plastic-stoppered centrifuge tube. For the tissue samples, it was sheared and thereafter 1.0 g of ground tissues (muscle and liver) was weighed into a 40.00 mL centrifuge tube. Ethyl acetate (4.00 mL) was added and the mixture was homogenized with a D-9 disperser (Miccra, Müllheim, Germany) for 10 sec at 16000 rpm. After centrifugation for 15 min at 4000 rpm, the supernatant was removed and transferred to a 15.00 mL plastic-stoppered centrifuge tube. Both the plasma and tissue sample extraction steps were repeated. The combined ethyl acetate extract was then evaporated to dryness in 40 ^°^C water bath pot under a gentle stream of nitrogen. The residue was dissolved in 1.00 mL of mobile phase solution and 0.50 mL hexane and then, was whirl mixed. After centrifugation for 20 min at 16000 rpm, the hexane layer was discharged. The water-based phase was filtered through a nylon centrifuge filter (0.45 μm). Aliquots of 20.00 μL were injected on the HPLC column.

**Data analysis. **The determination of pharmacokinetic parameters of florfenicol was done by Half-Life Calculator. The data obtained from tissue concentrations of florfenicol after the medicated feed and bath routes were analyzed by SPSS (version 19; SPSS Inc., Chicago, USA)‎. A *p* value less than 0.05 was considered significant.

## Results

**Fig. 1:**
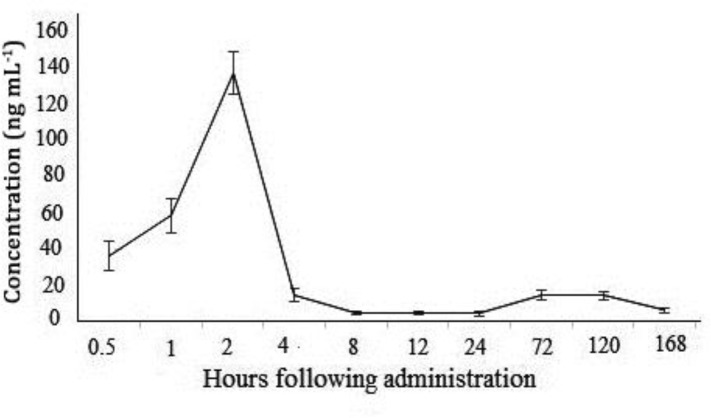
Florfenicol plasma concentration vs. time in common carp after a single oral dose.

The mean concentrations of florfenicol versus time in plasma of common carp after single oral dose administration were shown in Figure 1. Florfenicol was rapidly absorbed following oral administration. Plasma concentration of florfenicol after 30 min reached 36.13 ng mL^-1^ and maximum plasma concentration (137.02 ng mL^-1^) was gained at 2 hr. After this, the drug level declined rapidly and reached 6.45 ng mL^-1^, which was close to the limit of determination at 168 hr. The concentrations of florfenicol in plasma, muscle and liver samples after a single oral and bath administrations on ten consecutive days decreased over time after the initial increase (Table 1).

**Table 1:** The concentrations of florfenicol (mean ± SD) in plasma, muscle and liver samples after oral and bath administration.

**Medication route**	**Sample**	**Day 1**	**Day 7**	**Day 14**
**Oral treatment**	**Plasma (ng mL** ^-1^ **)**	35.84 ± 3.20 ^a^	24.21 ± 2.11^b^	Undetectable
	**Muscle (ng g** ^-1^ **)**	90.03 ± 4.06 ^a^	78.93 ± 3.07 ^b^	8.75 ± 1.28^c^
	**Liver (ng g** ^-1^ **)**	1352.44 ± 11.61^a^	694.07 ± 8.02 ^b^	20.03 ± 2.39 ^c^
**Bath treatment**	**Plasma (ng mL** ^-1^ **)**	11.40 ± 1.56	Undetectable	Undetectable
	**Muscle (ng g** ^-1^ **)**	496.02 ± 6.29 ^a^	56.98 ± 3.19 ^b^	6.30 ± 1.08 ^c^
	**Liver (ng g** ^-1^ **)**	1976.96 ± 10.83 ^a^	8.70 ± 1.47 ^b^	4.65 ± 1.31 ^c^

abc Different superscript letters mean there are significant differences between the concentrations in different days.

Concentrations of florfenicol in oral administration (medicated feed route) were significantly higher than concentrations of florfenicol in bath route in plasma and liver (*p < *0.05), but in muscle, the concentration of florfenicol was more comparable with oral treatment (*p < *0.05).

Some of the estimated pharmacokinetic and pharmaco-dynamics parameters for intravenous, bath and oral treatments with florfenicol are presented in Table 2.

**Table 2:** Pharmacokinetic and pharmacodynamics parameters for intravenous, bath and oral treatments with florfenicol.

**Pharmacokinetic parameters**	**Intravenous (median)**	**Oral gavage (median)**	**Bath treatment (median)**
**Time to reach peak concentration in plasma (hr)**	2	-	-
**Peak concentration in plasma (ng mL** ^-1^ **)**	137.02	-	-
**The elimination half-life (hr)**	21.40	-	-
**The volume of distribution at steady state (l per hr)**	0.30	-	-
**Drug concentrations in muscle (ng g** ^-1^ **), (day 1)**	-	496.02	90.36
**Drug concentrations in muscle (ng g** ^-1^ **), (day 7)**	-	56.98	78.93
**Drug concentrations in muscle (ng g** ^-1^ **), (day 14)**	-	6.30	8.75
**Drug concentrations in liver (ng g** ^-1^ **), (day 1)**	-	1352.44	1976.96
**Drug concentrations in liver (ng g** ^-1^ **), (day 7)**	-	694.07	8.70
**Drug concentrations in liver (ng g** ^-1^ **), (day 14)**	-	20.03	Undetectable
**Approximate withdrawal period (muscles)**	-	Over 14 days	Over 14 days

## Discussion

This study was done to evaluate the pharmacokinetic of florfenicol following oral administration and tissue residues of florfenicol in common carp. According to our results, florfenicol exhibited approximately similar pharmacokinetic properties in common carp as reported for other fish species.^5^^,^^6^^,^^9^^,^^14^ Several studies have performed on pharmacokinetic of florfenicol in different fish species. Oral administration is a common route of drug administration in fish.^4^^,^^6^

Due to the higher concentration of florfenicol after oral administration, it seems that florfenicol has been absorbed after oral administration better than after bath treatment.

Maximum level concentration in gavage route reached after 2 hr (137.02 ng mL^-1^). This shows that florfenicol rapidly absorbs in common carp. But the concentration of the drug, after 2 hr, declined and reached to 14.70 ng mL^-1^ in 4 hr. This finding indicates that florfenicol eliminates from plasma rapidly. The mean values of peak concentration and the time to reach peak concentration in the plasma of common carp in this study were lower and earlier than those in other species. These results indicate that the pharmacokinetics of florfenicol is affected by differences in fish species, drug administration and culture and experimental conditions. 

In Atlantic salmon, bioavailability or absorption of florfenicol in oral administration with a dose of 10.00 mg kg^-^^1^ was 96.50%, when water temperature was 10.80 ± 1.50 ˚C.^3^ In this fish species florfenicol was distributed to all organs and tissues with a dose of 10.00 mg kg^-1^ when the water temperature was 8.50 to 11.50 ˚C. Concentrations in muscle and blood were similar to serum concentrations, while fat tissue and central nervous system had lower concentrations. Only 25.00% of serum drug and metabolite concentrations were found in the brain.^18^

The results showed that florfenicol was detectable in both methods on day 14. The data indicate that withdrawal period for florfenicol in the muscles of common carp will pass after day 14. We propose one-month withdrawal period for this drug in common carp. Our results are in agreement with the common phenomena that drug clearance in fish is low and usually in several weeks low levels of drugs are generally found in the poorly perfused tissues such as muscle.

In addition to species-specific differences, the rate of elimination of florfenicol is also influenced by the water temperature at which fish are farmed as well as water salinity.^19^ Feng *et al*. observed that in tilapia reared in fresh water the primary route of excretion is the bile duct, with the possibility of an entero-hepatic circle (which increases the drug permanence in the body), whereas in the same species reared in sea water, the major route of elimination is through the gills (which leads to a more rapid excretion of the drug).^9^ It appears that even fish size can influence the depletion times. Bowser *et al*. estimated a shorter elimination time of florfenicol from edible tissue in smaller size fish.^20^ From the foregoing observations, it is clear that studies on florfenicol kinetic depletion have to be carried out in each different cultured fish species, in order to better define a safer use of the drug.

The volume of distribution at steady state (V_dss_) is a crucial pharmacokinetic parameter for indicating the diffusion of the drug in the body. When V_dss_ is greater than 1.00 L kg^-1^, florfenicol accumulates mostly in tissue. In common carp, the V_dss_ in plasma was 1.10 L kg^-1^. The pharmacokinetic interpretation of plasma florfenicol concentration data revealed that this drug was well distributed throughout the body in common carp.

In conclusion, the present study showed that florfenicol absorption in oral medication route was better than bath route in common carp. Absorption and elimination of florfenicol in common carp after single oral dose were done rapidly. Based on our study, the future use of florfenicol in the treatment of diseases in common carp may be taken into consideration. However, the research on the tissue distribution of drugs in aquaculture should be combined with the results of toxicological studies. For common carp, it may be better to choose another antibacterial agent with a higher bioavailability, more satisfactory distribution and shorter withdrawal time.
